# A Treatise on the Diseases and Special Hygiene of Females

**Published:** 1846-01

**Authors:** 


### Art. V.

*A Treatise on the Diseases and Special Hygiene of Females.* By Colombat de l'Isère. Translated from the French, with Additions, by Charles D. Meigs, m.d. &c.—*Philadelphia,* 1845. 8vo, pp. 720.

In the year 1838 M. Colombat de l'Isère published two volumes of a treatise on the Diseases of Women, and completed it by the publication of a third volume in 1842. He gives an account in this work of all the diseases of the impregnated and unimpregnated conditions, as well as of the puerperal state. Unfortunately, however, he possessed few qualifications, besides industry, for the performance of his difficult undertaking. His work is a mere compilation, often extremely ill-executed, and showing but little critical knowledge; the author's aim appearing to be that of quoting as large a number of writers as possible, while he pays but little regard to the comparative importance of their opinions.

Such being the character of this book, we cannot but feel surprised that a gentleman of Dr. Meigs's great experience should have thought it worth his while to engage in the wearisome occupation of translating it into our own language. He would seem, indeed, to have found it an irksome labour, if we may judge from the small amount of pains that he has devoted to the performance of his self-imposed task. We strongly suspect that Dr. Meigs has intrusted the main drudgery of translating to some inferior hand, thinking that the supervision of the proofs and the addition of notes to the text, would sufficiently justify the announcement of his name on the title-page as translator.

To show our readers how well Dr. Meigs is qualified to write on the subjects treated of in this volume, we will here notice some of the principal additions made by him to it—more especially such as are of a practical nature. We are sure that no one who peruses these additions will fail to express his regret that Dr. Meigs did not give us an original work of his own instead of a careless version of M. Colombat's.

*Epoch of puberty.* To the chapter on the "phenomena of puberty," Dr. Meigs appends a note on the epoch of puberty and the critical age of the females of the republic of Venezuela, founded upon the researches of Dr. Vargas of Caraccas. From these it appears that in 70 per cent. menstruation occurs from the age of 13 to 15 years, while the most common period for the cessation of menstruation is from the age of 45 to 48 years. Precocious menstruation is more common in the white than in the negro race.

*Menstruation.* We have been much surprised to read the following statement at p. 33. "It is well known, that in a great metropolis like Paris or Philadelphia, there exist multitudes of women who do not take the least precaution to prevent the blood of the menses from soaking through their clothes, and exposing their condition to the public eye in the street or in the market-place!" From a very extensive acquaintance with the habits of the poorer females of this metropolis (London), we are convinced that every precaution is taken by them to prevent such an indecent exhibition.

To the chapter on the cessation of the menses, Dr. Meigs adds a tabular statement, showing the age at which this function ceased in 183 females. His observations agree with those of M. Brierre de Boismont.

*Anatomy of the vagina.* At p. 48 he directs special attention to an omission in the original text in the anatomical description of the vagina, viz. that while the posterior and part of the lateral walls are inserted into the soft and distensible parts, as the perineum, the anterior and part of the lateral walls are firmly attached to the pubic arch; so that in labour, when the vagina becomes lacerated, it is the anterior or antero-lateral surfaces which give way, these not yielding so readily by reason of their firm attachment. In remarking upon the distensibility of the vagina, Dr. Meigs makes some judicious remarks upon the necessity of remembering this quality of it when the tampon is employed to arrest hemorrhage in early abortion or menorrhagia. The tampon he recommends "consists of portions of linen torn into squares of three or four inches, of which the pieces are successively introduced until the cavity is quite filled."

*Enlarged clitoris.* At p. 83, Dr. Meigs relates a case of enlarged clitoris, which was under the care of Dr. Norris, of the Pennsylvania Hospital. It occurred in a patient 36 years of age, had existed fourteen years, and commenced after she had received a blow on the part. It gave to the hand the sensation of indistinct fluctuation, and was not painful even when *smartly* compressed.

A common lancet introduced into the base of the tumour gave exit to about twenty-two ounces of a thick, blackish fluid, of the consistence of tar, perfectly inodorous, and very similar to retained catamenial fluid. After relating this case, the Doctor remarks, "so far as my knowledge extends, there is no example of blood detained for months and years in cavities, without undergoing decomposition, except when it is detained within the generative tissues."

*Malformations of the vagina.* At p. 91, he relates a singular case. The patient was in labour, and considerable delay and difficulty were experienced by the division of the vagina into two lateral halves by a septum which extended from the external orifice or os magnum to near the uterine extremity of the canal; the head of the child forced into the right half compressed the other against the left side of the pelvis. Dr. Meigs delivered the infant alive, "without accident or any untoward result." After the patient's recovery an accurate examination was made, when it was found that the septum had not given way during the distension of the right canal.

At pp. 95-99, some cases of congenital narrowness of the vagina are related, and in the remarks appended to these cases the translator takes occasion to recommend "simple graduated dilatation," and strengthens his recommendation by the advantages which follow gentle dilatation of the urethra in strictures of that canal; by the usual effects of the pains of labour on the cervix uteri, vagina and perineum, which he has "*familiarly*" noticed; by the gradual distension which the female urethra and rectum allow, the former permitting the index-finger, the latter the hand to be introduced. He strongly advises against the use of cutting instruments in such cases.

At p. 101 he records an interesting case of occlusion of the vagina after parturition, without any assignable cause. Fortunately Dr. Meigs was consulted before the cohesion which had taken place between the anterior and posterior wall of the vagina was very firm, and he therefore was enabled to separate them with the bulb of a probe, the patient losing not more than two or three drachms of blood. A second case is described, taken from the 'Philadelphia Practice of Medicine.'

*Absence of the womb.* To M. Colombat's chapter on this subject Dr. Meigs appends a note recommending that in cases of protracted emansio mensium, marriage should be postponed until after the menses have appeared, or until a competent inquiry has been made as to the fitness of the female to enter into the marriage compact, illustrating his observations by the recital of the case of a "handsome woman" by whom he was consulted, and who, although 22½ years of age, and married upwards of two years, had never menstruated. The mammæ were well developed, the pudendum amply supplied with hair, in fact there was perfect development of the sexual system, but total absence of menstruation. For two years and upwards the husband had failed in his attempts to accomplish sexual congress, when at length Dr. Meigs was consulted. Careful investigation discovered the vagina to be a cul-de-sac, scarcely two inches in length, and the uterus to be wanting. A similar case has lately fallen under our notice. Such cases, however, must be of rare occurrence, for when proceedings were instituted to obtain a nullity of marriage, no precedent was discovered in the records of the court.

*Prolapsus.* Dr. Meigs is of opinion that prolapsus uteri in many instances is occasioned by the floor of the pelvis, or tissues of the perineum losing their full power and energy, and especially by the weakness and loss of power of the levatores ani. He differs from M. Colombat in the opinion that "intense peritonitis" is induced as one of the complications of prolapsus, but regards it as simulated peritonitis, the diagnosis of which he admits may be very difficult.

*Polypus.* Dr. Meigs wisely cautions his readers against placing implicit confidence in a statement of Colombat when speaking of the diagnosis of polypus uteri—viz., that "polypus uteri is irreducible, and any attempt at reduction causes insufferable pain, while, on the contrary, prolapsus uteri is easily reducible, and its reduction gives great relief to the patient." Dr. Meigs most properly says we sometimes meet with examples of polypus uteri which are occasionally accessible, and at other times retire beyond the reach of the finger; for instance a pediculated polypus may be attached to the fundus, and may be forced partially through the os uteri by contraction of the parietes, and when such contraction ceases it will be again drawn into the cavity. (p. 126.) A case in point is at the present time under our care.

*Use of the pessary.* At pp. 136, 140, and 150, Dr. Meigs makes some judicious remarks on the pessary and its employment. We fully agree with him that "great abuses are to be met with in the prescription and use of this instrument," while "many persons are restored to health, or procure a tolerable state of health by its use." He urges that every case of prolapsus or procidentia uteri should be regarded as an affection of the vagina, and that the indication of cure should be "the restoration of this canal." He strongly advises against treating prolapsus by long continued rest in the horizontal posture, as it is apt to exhaust the "muscular force of the patient," "weaken the levatores ani," and "relax the perineum," which becomes nearly horizontal, or quite even with the tubera ischii; and, on the contrary, he recommends "exercise, air, and light, a nutritious diet, wine, and malt liquor," believing that in proportion as the general health improves, the local disorder will gradually lessen and finally disappear. When a pessary is indicated Dr. Meigs prefers the globular form of instrument, as it cannot become displaced by turning on its axis, and one made of silver, or silver washed with gold, of about two inches and a quarter in diameter. He advises "some time" to be taken in the introduction; for if it enter "too readily, or if it be not properly adjusted," it will be expelled at the first bearing down in defecation or in evacuating the urine. It is to be placed in the vagina beyond the constrictor muscle.

*Antiversion and retroversion.* At p. 159 we find some remarks on antiversion and retroversion. Dr. Meigs states that he has seen but one decided case of antiversion, although he has met with many of retroverted uterus. In many points he differs from M. Colombat in his account of these displacements. He maintains that women who habitually permit the bladder to be considerably distended will in the end cause the round ligaments to be so relaxed and stretched as to be perfectly useless. He relates the case of a lady whose round ligaments are so useless that the womb falls over into the hollow of the sacrum from the slightest effort, so that on many occasions he "has had to *reposit* it." He believes in the contractility of these ligaments.

In cases where extreme difficulty has been experienced in the attempts to reduce a retroverted womb, Dr. Meigs directs the patient to be placed upon her knees, with her thighs at right angles to the bed, and the sternum in contact with the mattress. By such a position the weight of the viscera is taken off, the power of "tenesmic" resistance is wholly removed, and the reposition of the womb is favoured. We perfectly agree with Dr. Meigs that this mode of preventing the "tenesmic power" is preferable to bleeding "ad deliquium," as recommended by Dewees. Few cases will resist reposition under such circumstances, and when the attempts at the restoration fail, it will in most instances be caused by adhesions.

*Obliquity of the uterus.* At p. 175 the translator protests against the statement of M. Colombat that "right lateral obliquity takes place in ninety-nine cases in a hundred," and affirms that "left lateral obliquity is not less frequently met with than that of the right side;" we must beg leave to differ from Dr. Meigs, and maintain that right lateral obliquity is more frequently met with than left lateral obliquity, and we must express our astonishment that a contrary opinion is entertained by a physician of so large experience.

*Inversion of the uterus.* To the chapter on "Inversion of the Womb," Dr. Meigs appends two most interesting cases of reposition of the womb after inversion, and in which both the women afterwards became pregnant. These extraordinary cases are confirmed by the testimony of several other respectable practitioners.

*Labour complicated with enterocele.* At p. 207 a most instructive case of labour, complicated with vaginal enterocele is recorded; it had lasted fourteen hours and a half. The hernia was reduced while the patient was on her left side, by the fingers of the right hand introduced into the vagina. After the reduction, four pains expelled the child.

*Prolapsus vaginæ.* At p. 211 a case is mentioned where the vagina prolapsed to the extent of five inches, and was as large as a man's arm.

*Support of the perineum.* In a short note at p. 218, the Professor gives some valuable advice as to the mode in which the perineum should be supported during labour. He properly directs that the perineum should be so pressed that the head should be made to turn upwards in front of the symphysis in proportion as it emerges more and more from the pelvis in the direction of the circle of Carus. Attention to this rule would prevent many perineal lacerations.

*Prurigo vulvæ.* At p. 271 Dr. Meigs speaks highly of the efficacy of the following lotion in purigo of the vulva. R. Sod. biborat. ʒss, morphie sulph. gr. vj, aq. rosæ ℥ vj; m. ft. lot. It should be applied three times a day by a piece of sponge or linen, the surface being previously washed with tepid water and then well dried.

*Abscess of the labia.* To the chapter on phlegmonous inflammation of the labia the translator appends a caution, guarding the young and inexperienced practitioner against mistaking a vulvar enterocele for a supposed abscess.

*Physometra.* Professor Meigs does not believe that the disease termed physometra does or can really exist. In one case only has he known gas discharged from the uterus, and that was during an embryotomic operation, the placenta which was subsequently removed being putrid, black and emphysematous. He does not believe that "the inner walls of the womb" have the power "to excrete gaseous fluids." Neither will he admit that the os or cervix uteri can become "air-tight." He ridicules the opinion of Dr. Waller, who believes the air to be secreted by the "*menstruating membrane.*" He regards all cases of supposed physometra to be cases of chronic tympanitis.

*Polypus uteri.* At p. 388 Dr. Meigs differs from M. Colombat in his views of the sensibility of polypus. M. Colombat having stated that the existence of nerves, although not demonstrable, may be proved by the pain resulting from the constriction of their pedicles. Dr. Meigs, believing the tumour to be insensible to pressure, supposes that the constitution "disturbs or distracts the sensitive part upon which it sits." Its organization and nutrition, prove that it has blood-vessels, and such vessels must have accompanying nerves. At p. 396 a case is reported in which a large polypus, weighing eleven ounces, was expelled from the uterus two days after the delivery of a fine healthy male child. Dr. Meigs is of opinion, and we agree with him, that the development of the tumour must have proceeded at a great rate during the latter months of gestation.

*Ovarian abscess.* At p. 412 the translator relates the case of an "ovarian abscess" occurring in a young woman after her third confinement. It pointed in the lower part of the left iliac region, and on a puncture being made with a lancet, a pint of pus was discharged. This seems to have been a case of common pelvic abscess after delivery. In the next page Dr. Meigs combats the idea of treating as "ovaritis" those inflammations of the ovary which are met with during the progress of puerperal fever. He admits that deposits of pus are sometimes found beneath the ovarian peritoneum, but he regards it as "disparaging the idea of a puerperal fever to call it an ovaritis."

*Extirpation of the ovary.* Dr. Meigs fully concurs with M. Colombat in his disapprobation of operations for the extirpation of the diseased ovary, regarding such operations as not to be "justified by the most fortunate issue in any ratio whatever of the cases."

*Treatment of ovarian diseases.* Dr. Meigs gives some valuable advice as to the best means of checking ovarian disease when discovered early. "The diet" he says, "should be regulated, it should neither be too spare nor too nutritious; the clothing should be warm, especially about the pelvic region and lower extremities; the bowels should be kept in a soluble state; the patient should take a bath at 97° three times a week, and go from her bath to her bed; leeches sufficient to take four or five ounces of blood should be applied to the left groin, over the round ligament, four or five days before each menstrual period. She should confine herself to the house for three or four days of the period of return, and for some months she should take the hydriodate of potassa in three to five grain doses, thrice a day."

*Menstruation.* At p. 459 Dr. Meigs condenses and recapitulates the opinions of Lee, Negrier, Gendrin, and Raciborski, with respect to the changes produced in the ovary itself at the period of the catamenial flow; and is of opinion that the "hyperemic affluxion," which is "coincident with the stated periodical, monthly completion of a Graafian vesicle" and "which hyperemia relieves itself by the menstrual discharge," gives us "the key to all the derangements of the catamenial office, not dependent upon obturation(?) or some sudden shock and diversion of the nervous power to other directions."

*Dysmenorrhœa.* Dr. Meigs approves of Dr. Mackintosh's plan of dilating the cervix uteri, which he says has been productive in his hands of advantage in several instances.

*Metrorrhagia.* In this disease Dr. Meigs recommends a decoction of the roots of the common black currant, ripe, and the roots of the dewberry. A handful of each is to be boiled with two quarts of water, and after straining the liquor, the patient is to take a cupful occasionally for a dose.

*Chlorosis.* In speaking of the diagnosis of chlorosis, Dr. Meigs remarks that "every instance of anæmia is not discoverable upon inspection of the tint of the skin, nor a survey of the state of the patient as to her embonpoint," for some patients "grow fat during the malady." In such cases Dr. Meigs "tests the state of the lungs by asking the patient to make several forced inspirations, in order to discover whether the capacity of the lungs for atmospheric air are at all lessened by disease," and if "she appear to be able to inhale fifty or sixty cubic inches at an inspiration." Dr. Meigs would conclude that the air-cells are free, "and this view may be confirmed by percussion and auscultation of the chest." In reference to the frequency of the pulse, Dr. Meigs says, that whilst in a state of rest it may be great, as at 70, 80, or 90 beats in a minute; but if the patient be requested to walk to the head of the stairs and returns immediately to her seat, she will, if anæmic, be found to have the pulse greatly accelerated and beating in the most *troublous* manner, to the number of 120, or even 160 pulsations per minute, while her respirations may amount to 40 or even 60 per minute. In the treatment of chlorosis, Dr. Meigs recommends the citrate of iron, combined with the sulphate of quina, the following is his formula:

Citrate of iron 2 drs.
Sulphate of quinine, dr. ss.
Water one ounce. Mix

From 20 to 30 drops to be taken for a dose in syrup and water.

He advises with Raciborski that the draughts should be taken within half an hour after the meal, so that it may be carried with the chyle along the course of the bowels.

*Impotence and sterility.* Dr. Meigs relates the case of a lady who died with symptoms of strangulated hernia, and who upon examination was found to have a knuckle of intestine which had passed under a band within the pelvis, one of numerous bridles which had been produced many years before, when she was 13 years of age; and had suffered from an attack of peritonitis, to which she had nearly fallen a victim. Her fallopian tubes were adherent, so as to render their physiological function impossible, and although married for several years, she had not conceived. Sterility in some cases Dr. Meigs supposes to depend upon the plugging up of the cervix uteri, by a thick, translucent, transparent, or opaque mass of mucus, in amount about a teaspoonful, cohesive and ductile, so that it may readily be drawn from the cervix upon the point of a sponge. He supposes it to be produced by the mucous follicles just within the os uteri. For its removal or treatment, he recommends the occasional cauterization of the canal of the cervix with nitrate of silver or a pencil of sulphate of copper, astringent injections, baths, a long voyage, and change of climate.

*Abortion.* The translator's notes to the chapter on abortion, although brief, are valuable; he is of opinion, that in the majority of cases of abortion, the cause depends upon the death of the embryo. He alludes to the case of a lady who, at the middle of the third month, had uterine hemorrhage to the amount of more than twenty ounces without losing the embryo, and who was delivered of a healthy child at full term. We perfectly agree with Dr. Meigs as to the difficulty of making a correct diagnosis in cases where pregnancy has occurred, and the death of the embryo after a time has taken place, the ovum being retained in the womb for several months. Our opinion must be formed upon the previous history, especially the dates of the menstrual periods, the form and size of the womb as determined by external and internal examination, the condition of the cervix and os uteri, together with the present and past condition of the mammary glands and areolæ.

In cases of repeated miscarriages, Dr. Meigs has a high opinion of the plan of treatment recommended by the late Dr. Physick, viz. the use of an anodyne enema, consisting of a wineglassful of starch mixed with forty drops of laudanum, introduced every night until quickening takes place. Dr. Meigs does not approve of removing the placenta after abortion by force, stating most justly that such a practice is both unnecessary and dangerous. He advises the use of the tampon in all severe hemorrhages from abortion up to the period of four months to four months and a half.

*Disease of the lungs in pregnancy.* Some very judicious practical remarks are added by the translator to the chapter on pregnancy. In speaking of *dyspnea* in pregnant women, he says such a complication deserves close and most careful scrutiny. Every part of the chest should be closely auscultated, the capacity of the lungs for air should be tested, by causing the patient to make a forced inspiration. "A patient attacked with inflammation of the lungs is not very easily cured, if in an advanced stage of pregnancy, until after her delivery shall have taken place." He further remarks that many women complain of difficult respiration in pregnancy and of violent palpitation and unusual disorder of the heart, in consequence of their having become anemic during the last months or weeks of gestation. In cases where the anemia proceeds to an aggravated state, the consequences are often most distressing, the patient is liable to troublesome and even very dangerous effusions of serum into the chest, pericardium, and abdomen. A mistake on the part of the practitioner would be most unfortunate for the patient by misleading him in his treatment. He must "carefully discriminate between the effects of a pure inflammation and those often similar ones that arise out of the feeble and irregular innervation proceeding from the state of the blood."

*Flooding.* At p. 612, Dr. Meigs speaks most forcibly of the impropriety of using the tampon in the hemorrhages of advanced pregnancy; and further disapproves of this remedy in placenta prævia. He lays great stress upon the uses and value of position, as a means of diminishing or suppressing the flooding; the woman should lie upon the bed, with the head but slightly raised, while the shoulders are upon the same plane with the rest of the trunk. In cases of copious hemorrhage, where the pulse is full and bounding, where there are heat of skin and flushing of the face, he advises bloodletting, provided the general state of the patient's health will warrant such a proceeding; but in determining this we are to discriminate between the cases which occur from a "*nisus hæmorrhagicus*" and those simple effusions of blood which proceed from an accidental detachment of a portion of the placenta. He condemns frequent examinations, as both mischievous and useless. Some brief but valuable directions as to the best plan of arresting hemorrhage are given; he speaks, and we think justly, of the great value of opium, at the same time he affirms that the patient is not safe until the uterus is emptied, and its contraction secured. Some practical remarks are further made on that form of hemorrhage which supervenes an hour or two after delivery, arising from the dilatation and expansion of the uterus. Dr. Meigs forcibly insists on the introduction of the hand into the uterus to remove the coagula and excite contraction, and guards his readers against packing the napkins too tightly against the os internum, for thus they act as a tampon, stop the blood in the vagina, which first, and afterwards the uterus, becomes filled.

*Varicose veins.* At p. 623, after affirming that the evils of varices may in general be obviated by the use of the roller and other remedies, the translator states that he has sometimes seen dangerous and even fatal crural phlebitis supervene after labour, taking its rise in the diseased and distended veins of the leg.

*Convulsions.* At p. 626, Dr. Meigs suggests that eclampsia is more or less intimately connected with œdema of the extremities, especially in primiparæ. At p. 636 he expresses his confidence that if cases of puerperal convulsions are judiciously treated, the mortality will not exceed 14 or 15 per cent., Colombat's experience being that about one half of the cases prove fatal. Dr. Meigs agrees with Colombat in his treatment of such cases by a bold and vigorous employment of the lancet, but differs from him in the administration of opium, where full bloodletting has been premised, and after the bowels have been unloaded by active enemata. He agrees with Puzos that should bloodletting and other remedies fail in arresting the convulsions, the female should be delivered as soon as possible. He agrees with Colombat and most other pathologists, that the brain when examined after death usually presents no lesions to explain the violence of the disorder. We cannot agree with the translator in his note at p. 645, that eclampsia never occurs in anemic patients or in patients who have been drained by copious hemorrhage.

*After-pains.* At p. 645 Dr. Meigs most properly suggests the necessity for the greatest caution being used in deciding what is and what is not after-pain; in doubtful cases, his practice is "to lessen the force of the circulation by a bloodletting proportioned to the exigency."

*Puerperal fever.* From p. 665 to p. 682 there is a long note on the subject of puerperal fever. Dr. Meigs appears to be throughly conversant with the disease in its various forms, its pathological appearances, and the several modes of treatment. He differs from Fergusson, Collins, and others who regard puerperal fever as "something over and above the local disease," "something beyond inflammation of tissues," and states "that phlebitis of the recently discharged womb alone," "gangrenous inflammation of the inner pains of the uterus," or "inflammation of the peritoneum," all or either is sufficient to produce all the "frightful rapidity which attends the disease," and to cause "the rapid and sudden overthrow of all the functions." He extols most highly the small but excellent volume published by Dr. Gordon of Aberdeen in 1795, and like this physician places more confidence in bloodletting at the commencement of the disease than in any other remedy. Gordon insisted upon 24 ounces of blood being drawn "in the early stage, within twenty-four hours of the attack." Meigs is unwilling to adopt this quantity as "an universal rule," since "the same point is attainable in some by 24 ounces, in others by not less than 30, and in others by 12 or 15." He is guided by the "pulse, breathing, cessation of pain, voice, gesture, *decubitus,* physiognomonic expression, and general sensations of the patient." He differs from Dr. Collins of Dublin, (although professing the greatest respect for his opinions,) in the preferableness of leeching in the epidemic forms of the disorder, and especially on the ground that "the uterine and ovarian circulation is in nowise directly related to the circulation in the skin," and strengthens his remarks by referring to the observations of M. Baudelocque fils, in his 'Traité de la Péritonite puerperale.'

*Swelled leg.* At p. 683 he very properly corrects an error of M. Colombat, who states that in "milk-leg" the pain "commences in the groin and leg" and the swelling extends "from above downwards." Dr. Meigs says, and we think justly, that the swelling "is in a great majority perceived first in the calf of the leg, which becomes painful, hard, and swollen before the woman suspects that she has any pain at the groin or in the thigh."

---

